# Late-Presenting Eosinophilic Granulomatosis with Polyangiitis as Cause of Pulmonary Fibrosis: A Case-Based Review

**DOI:** 10.31138/mjr.161023.lpe

**Published:** 2024-02-12

**Authors:** Juan Felipe Coronado-Sarmiento, Juan Pablo Coronado-López, Eduardo Tuta-Quintero, Claudia Marcela Mora, Viviana Mayor

**Affiliations:** 1 Faculty of Medicine, Universidad de la Sabana, Chia, Colombia,; 2 Health Sciences Faculty, Pontificia Universidad Javeriana, Cali, Colombia,; 3 Internal Medicine Department, Universidad de la Sabana, Chia, Colombia,; 4 Internal Medicine Department, Universidad del Bosque, Bogota, Colombia

**Keywords:** eosinophilic granulomatous vasculitis, ANCA-associated vasculitis, pulmonary fibrosis, interstitial pneumonia

## Abstract

**Introduction:**

Eosinophilic granulomatosis with polyangiitis (eGPA) is a necrotising vasculitis of small and medium calibre vessels, which affects mostly patients in their fourth to sixth decade of life, and it is a very uncommon aetiology for pulmonary fibrosis.

**Clinical case:**

A Hispanic 72-year-old female patient presents with a history of lower extremities pain, paraesthesia, oedema, and occasional macroscopic haematuria. During her hospitalisation, the patient presents, and images showed findings compatible with pulmonary fibrosis and alveolar haemorrhage, which require a biopsy, establishing the diagnosis of an eGPA.

**Discussion:**

eGPA is a low-incidence autoimmune vasculitis, with a high number of phenotypes which explain the broad clinical spectrum, but recent advances has helped to understand the physiopathology and its link with other conditions like pulmonary fibrosis.

**Conclusion:**

Early diagnosis and management of this condition is mandatory because it is the only factor that change the outcome of the patients.

## INTRODUCTION

Eosinophilic granulomatosis with polyangiitis (eGPA) is a necrotising vasculitis of small and medium calibre vessels, associated with the presence of antineutrophil cytoplasmic antibodies (ANCA).^1,2^ The eGPA has a prevalence between 4–22 cases per one million people, in their fourth to sixth decade of life, but the incidence of this condition has been described even in older people, without female/male predominance. In the general population, the incidence is between 0.5 to 4.2 cases, but it increases up to 64.4 cases per one million people in asthmatic patients.^1–3^

This entity is characterised by eosinophilia over 10% in the total leucocyte count, bronchial asthma, and necrotising vasculitis of small and medium calibre vessels, but there could be other manifestations including cardiovascular, renal, and neurological, however, these displays represent a minor proportion of this condition.^1–4^

Currently, pulmonary fibrosis due to eGPA is relatively rare, because the lack knowledge available regarding its physiopathology, diagnosis, and treatment; these fundaments are necessary for improving the clinical outcome of these patients.^5^ We present the case of a 71-year-old patient with neurological, pulmonary, and renal compromise due to eGPA.

## CLINICAL CASE

A Hispanic 72-year-old female patient presents to the emergency room with a 6-month history of progressive lower limb pain, sensation of paraesthesia, oedema, and limitation to her gait. During the review of systems, the patient reports occasional macroscopic haematuria without any urinary irritative symptom. In her medical records it was relevant one year ago she was diagnosed with preserved ejection-fraction heart failure in management with furosemide 20mg per day and metoprolol tartrate 50mg per day.

Her initial physical examination showed vital signs within normal values, cardiac auscultation did not show any pathological findings, but lung auscultation showed Velcro sound in both pulmonary bases, grade I soft oedema in both inferior extremities, and her neurological examination showed diminished strength in both, flexion, and extension in her toes bilaterally, right knee, right hip, left elbow, and left shoulder. There were found absent in both extremities the Achillean and tricipital reflex. She also had diminished the superficial sensitivity and she presented allodynia. Initial workup in the emergency room showed altered renal function, moderate proteinuria, anaemia, eosinophilia, and positive ANCA, with further analysis showing positive anti-myeloperoxidase antibodies (**Table 1**). Based on this, she was admitted for beginning with replacement renal therapy and further studies to rule out immunological or haematological disease.

**Table 1.** Studies performed on the patient.

**Test**	**Result**	**Reference Value**	**Reference unit**

Creatinine	9.95 mg/dL	0.6 - 1.1	mg/dL

Ureic nitrogen	85.3 mg/dL	8 - 20	mg/dL

Na^+^	134	135 - 150	mEq/L

K^+^	5.31	3.5 - 5	mEq/L

Cl^−^	98	96 - 106	mEq/L

Mg^++^	2.08	1.7 - 2.2	mg/dL

Ca^++^	9.2	4.8-5.6	mg/dL

P^−^	7.36	4 - 7	mg/dL

TSH	0.64	0.3 - 4.7	mUI/L

PTH	58.7	10 - 55	pg/mL

LDH	193.4	140 - 280	UI/L

Coombs direct rest	Positive	Negative	-

Glycemia	84	60 - 100	mg/dL

Albumin	3.94	3.4 - 5.4	g/dL

**Immunoglobulins**			
Light chains kappa	4.95	3.3 - 19.4	mg/L
Light chains lambda	3.30	5.71 - 26.3	mg/L

**Cell Blood Count**			
Haematocrit	20.9	36 - 48	%
Haemoglobin	6.9	12 - 16	g/dL
MCV	96.3	80 - 100	fL
Leucocytes	7.69	4 - 10	10^3^ x mL
Neutrophiles	4.33	2 - 4	10^3^ x mL
Lymphocytes	1.69	1 - 2	10^3^ x mL
Reticulocytes	24	0–2	%
Eosinophiles	1.67 / 21%	<5 / <1	10^3^ x mL / %

**Uranalysis**			
pH	6.0	5 - 7	-
Density	1.10	1.005 - 1.030	-
Proteins	75	0 - 14	mg/dL
Leukocyte esterase	Negative	Negative	-
Nitrites	Negative	Negative	-
Epithelial cells	2	1 - 2	Por campo
Red blood cells	10	0 - 1	Por campo
Proteinuria 24 horas	580	<80	mg/24 horas
Volume over 24 hours	400	800–2000	mL/24 horas

**Ferrokinetic profile**			
Fe++	39.8	60 - 170	mcg/dL
TSAT	24	30	%
TIBC	160	250 - 450	mcg/dL
Ferritin	632	12 - 150	ng/mL

Peripheral blood smear	Diminished red blood cells with normal size and colour, significant eosinophilia.	-	-

**Arterial Blood Gas**			
FiO2	36	-	%
pH	7.46	7.35 - 7.45	-
PCO2	25.8	38 - 42	mmHg
PO2	58.1	75 - 100	mmHg
HCO3^−^	21.3	22 - 28	mEq/L
SatO2	92	92 - 100	%
BE	3	+/− 2	mEq/L
PaO2/FiO2	161	>300	mmHg

ANAs	1/160	< 1/40	Dilutions

FR	5	< 15	UI/mL

Anti-Ro	Negative	Negative	-

Anti-La	Negative	Negative	-

Anti-Sm	Negative	Negative	-

Anti-SLC70	Negative	Negative	-

ANCA’s	Positive	Negative	-

Anti-MPO	23.2 (Positive)	<1	UI/mL

Anti-PR3	1.5 (Negative)	<1	UI/mL

Anti-DNA	Negative	Negative	-

Anti-MBG	Negative	Negative	-

**Complement**			
C3	105	88 - 201	mg/dL
C4	23	15 - 45	mg/dL

Elisa VIH	Negative	Negative	-

HbsAg	0.04	<1	UI/mL

Anti-HCV	0.04	<1	UI/mL

CMV PCR	Negative	-	-

Respiratory film array*	Negative	-	-

Mycobacterium tuberculosis	Negative	-	-
PCR			

Mycobacterium tuberculosis on pulmonary tissue biopsy	Negative	-	-

Pneumocystis Carinii on pulmonary tissue biopsy	Negative	-	-

Na+: Sodium; K+: Potassium; Ca++: Inorganic Calcium; Mg++: Magnesium; P−: Phosphorus. Cl−: Chlorine; TSH: Thyroid-stimulating hormone; PTH: Parathyroid hormone; LDH: Lactate dehydrogenase; Fe++: Iron; TIBC: Total iron binding capacity; TSAT: Percentage of transferrin saturation; FiO2: Fraction of inspired oxygen ; PCO2: Partial pressure of carbon dioxide; PO2: Partial pressure of oxygen; HCO3−: Bicarbonate; SatO2: Oxygen saturation; BE: Base/excess; ANA: Antinuclear antibody; RF: Rheumatoid factor; Anti-Ro: Antibody anti-rhodopsin; Anti-Sm: Antibody against Smith factor; Anti-SLC70: Antibody against cytokeratin; ANCA: Antineutrophil cytoplasmic antibody; HIV: Human immunodeficiency virus; HbsAg: Hepatitis B virus surface antigen; Anti -HCV: Antibodies against hepatitis C virus; Anti-MPO: Antibodies against myeloperoxidase; Anti-DNA: Antibodies against deoxyribonucleic acid; Anti-MB: Antibodies against glomerular basement membrane; Anti-PR3: Antibodies against protease type 3; PCR: Polymerase chain reaction;

* BIOFIRE® FILMARRAY® Pneumonia Panel plus which include Acinetobacter calcoaceticus-baumannii complex, Enterobacter cloacae, Escherichia coli, Haemophilus influenzae, Klebsiella aerogenes, Klebsiella oxytoca, Klebsiella pneumoniae group, Moraxella catarrhalis, Proteus spp, Pseudomonas aeruginosa, Serratia marcescens, Staphylococcus aureus, Streptococcus agalactiae, Streptococcus pneumoniae, Streptococcus pyogenes, Legionella pneumophila, Mycoplasma pneumoniae, Chlamydia pneumoniae, Influenza A, Influenza B, Adenovirus, Sars-COV2, parainfluenza virus, syncytial respiratory virus, rhinovirus, human enterovirus, metapneumovirus

**Figure 1.**
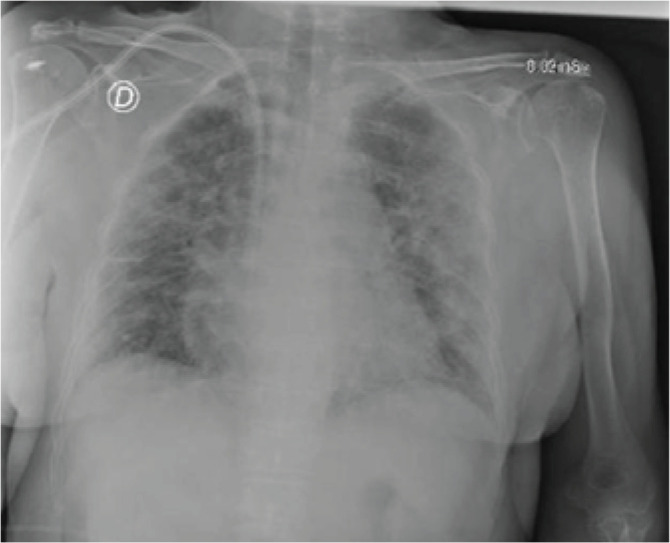
Anteroposterior chest X-ray (AP), showing mild cardiomegaly, symmetric pulmonary vasculature, and basal subpleural reticulation, with mixed opacities in both pulmonary fields in a ground glass pattern, most of them, occupying the alveolar spaces, probably as result of alveolar haemorrhage.

**Figure 2.**
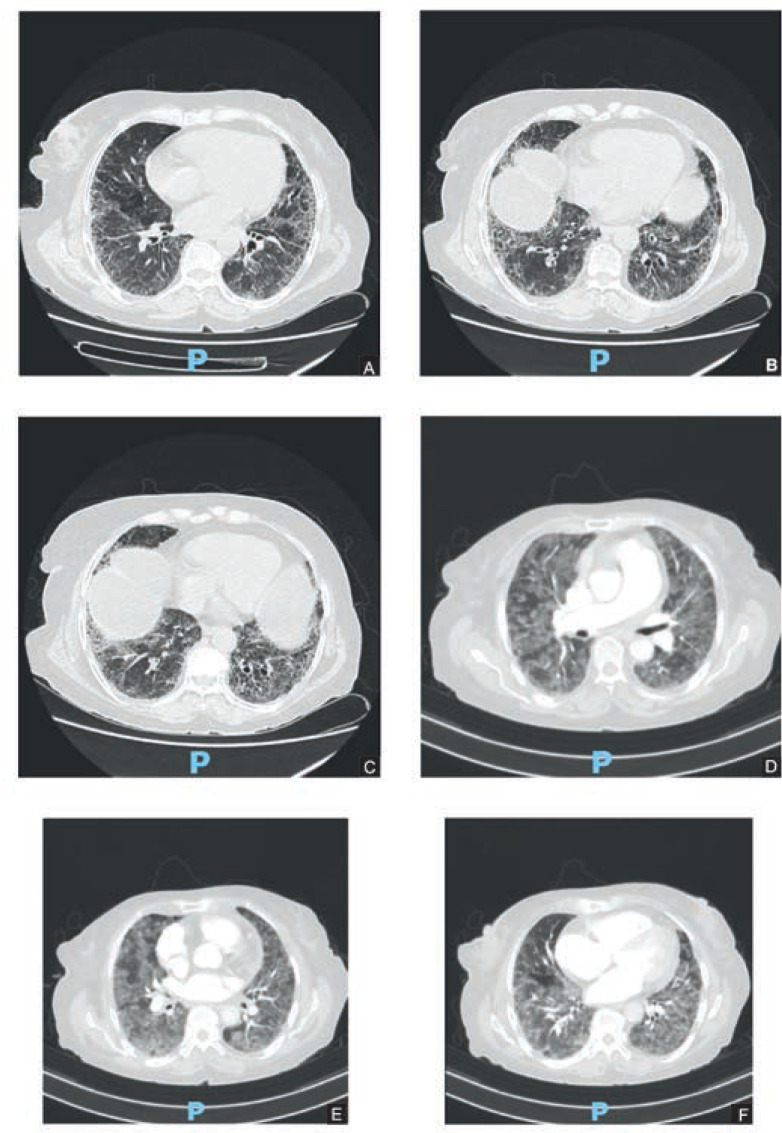
High-definition chest tomography (HRCT). **(A–C)** Increase in the peribronchovascular interstitial thickening interstice, thickening of the interlobular septum and honeycomb transformation, mostly in the mid and basal pulmonary fields, as well, bronchiolectasis caused by traction, suggesting usual interstitial pneumonia (UIP), along with mild cardiomegaly and increased diameter of the pulmonary trunk, reaching 37 mm. **(D–F)** HRCT taken after the deterioration of the patient, showing ground glass opacities along with interlobar septal thickening. In these boxes, there are no changes in the vascular portions or in the cardiomegaly measurement.

During her clinical evaluation, her lungs were characterised to ruled out any lung condition, however, later in her hospitalisation she presented tachypnoea, along with haemoptysis and supplementary oxygen need. Initially a chest X-ray was performed, which only showed alveolar opacities with mild cardiomegaly or any vascular congestive signs (**Figure 1**). A high-resolution tomography chest tomography (HRCT) was performed for characterising these findings, which showed a usual interstitial pneumonia (UIP) presented as fibrotic interstitial lung disease (**Figure 2[D–F]**). The clinical correlation with these features led to the clinical suspicion of an alveolar haemorrhage; for this reason, the patient was transferred to the intensive care unit (ICU), infectious processes were ruled out, and previous antiparasitic therapy and isolation, management with methylprednisolone pulses was performed, with improvement in the chest discomfort and her saturation values. Several days after, a bronchoscopy was performed in order to confirm the diagnosis, and the biopsy showed an important interstitial infiltrate of eosinophils, leucocytoclastic vasculitis with necrosis, fulfilling the diagnostic criteria^2^ of eosinophilic granulomatosis with polyangiitis. After this, immunomodulator management was initiated, and rituximab was chosen considering the renal function of the patient. Four weeks after the therapy was started the patient had a good response and was discharged without supplementary oxygen therapy, but with inter-day haemodialysis.

## DISCUSSION

The eGPA is an autoimmune disease with a low incidence, where most of the cases are diagnose around forty-eight years old^6^, and the diagnosis is mostly based on the clinical features, including classically rhinitis or asthma, with eosinophilia in the cell blood count and later, necrotising vasculitis findings.^7^

Most vasculitides are the result of immune-derived mechanisms, but due to a combination between genetic predisposition, epigenetic factors, environmental agents, and self-immune response.^8,9^

It has been documented that either rhinosinusitis or asthma must be present to diagnose eGPA, but recent data has shown this feature in adults depends on the environmental conditions,^6,7^ which can explain why our patient did not have this condition; however, in the adulthood these conditions have mostly environmental influence,^8^ explaining the absence of these conditions in our patient. It does not rely only on this factor, however: others such as expressivity and penetrance of the of the loci that code for HLA-DQ, which influence the autoimmune presentation of this disease, might explain its probable late development.^7,8^ Nevertheless, the mutations are not limited only to an entire locus, as point mutations like the one affecting the thymic stromal lymphopoietin (TSL), a cytokine derived from epithelial cells, responsible for the regulation of T cells and usually stimulated by pollution, bacteria and viruses; when it is mutated, it is associated with ANCA-negative eGPA, the eosinophilia and asthma severity,^8^ which our patient should not have based on the absence of this typical feature of the disease.

ANCA are autoantibodies against the neutrophil cytoplasm, expressed in the lysosomes of the monocytes and within the granules of the neutrophils, but there are only two with clinical relevance, the anti-myeloperoxidase (anti-MPO) and the anti-proteinase 3 (anti-PR3), because these are found in up to 50% of the patients with eGPA, but with great sensitivity and specificity values.^8^

ANCA-positive antibodies have been related with a different of infections including fungal, viral, bacterial, and protozoal, but also with subacute bacterial endocarditis and cystic fibrosis. It is theorised that most of these infections can lead to the development of ANCA antibodies due to molecular mimicry and can persist over the time due to T and B cell stimulation caused by microbial superantigens.^7,8^ For example, it has been proven that some patients with ANCA-associated vasculitis create antibodies against the lysosome associated membrane protein-2 (LAMP-2), which is found in the neutrophils. This protein has a close similarity with the adhesive protein FimH, expressed on the surface of the gram-negative bacteria, and these antibodies, both LAMP-2 and FimH can produce glomerulonephritis in animal models.^7^

The granulomatous inflammation and ANCA antibodies production seen in the eGPA are product of the abnormal response of the lymphocytes, both T and B cells. T cells are found active in peripheral blood of patients with eGPA, even if the disease is in remission, and these activation markers are related with its activity.

The immune implication of the ANCA include suppression of the regulatory T-cells and increasing the pro-inflammatory state of these cells, also leading to B-cell hyperreactivity.^9^ Recently, it was found that anti-MPO and anti-PR3 are important in the physiopathology of pulmonary fibrosis (PF), which can lead to explain a lot of previously named idiopathic cases. PF could be aggravated by the deficiency of the neutrophil extracellular traps (NET), which are nucleic acid residues, often joined to histones, structural proteins, or enzymes (myeloperoxidase or proteases) released in the extracellular space by active neutrophils^10^; with the function of neutralising some pathogens and regulating the inflammation process, but, when they are deficient could lead to autoimmune, metabolic, or neoplastic processes,^11^ which, along with the lymphocytes hyperreactivity^9^ result in the ANCA formation, therefore precipitating a vicious cycle that might explain the inflammation/scarring process of the PF.

Regarding the clinical features of eGPA, most of the patients present initially a difficult-to-manage asthma or rhinosinusitis,^1,7^ which usually improves after the incorporation of steroids in the treatment, controlling most of the symptoms for a period between 3 to 9 years. After this period, the clinical features derived from the necrotising vasculitis will appear, but only up to 30% of the patients will develop symptoms.^8,9,12^ These features include heart disease, with a broad spectrum of pathologies including myocarditis^13^ (main feature), arrythmias, coronary disease, constrictive pericarditis and restrictive cardiomyopathy.^6,12^

Regarding the pulmonary features, findings are heterogeneous, depending on the several factors, including severity of the disease, type of antibodies involved, and genetics.^12^ Initially, patients are evaluated with a chest X-ray where ground glass opacities (GGOs) are the most common findings, but not limited to them, interstitial thickening, and consolidations can be seen. In the high-resolution chest CT findings are GGOs, lung nodules, segmental bronchial wall thickening, septal lines, consolidations, lobar bronchial wall thickening, and different degrees of pulmonary fibrosis including bronchiectasis, honeycomb pattern and reticular septal thickening often with subpleural and posterior basal predominance.^4,12,13^ The main symptom is dyspnoea, followed by cough, haemoptysis, or constitutional symptoms.^13^ Despite the importance of establishing the aetiology of the PF, there was no significant difference either with the management or outcome with patients with idiopathic PF, with a 50–60% survivor rate over 5 years.^13,14^

Renal compromise affects up to 25% of the patients with EGP,^12^ and it is one of the main independent factors of mortality of this condition,^4^ considering that if the debut of this condition implies a glomerular fraction rate <50mL/min, mortality rises to 50% within the first 5 years. The histological diagnosis is usually no needed because eGPA does not have a particular pattern, because the spectrum includes from a pauci-immune occupation of the Bowman′s capsule to necrotising glomerulonephritis,^4^ with or without features of other conditions including IgA nephropathy, eosinophilic nephropathy, or focal and segmental glomerulosclerosis.^4,12^

Nervous system affection^15,16^ has different symptoms most of them due to a multiple mononeuropathy, according to the foci of eosinophilic vasculitis.^14^ These symptoms include painful paraesthesia, allodynia, crawling sensation mostly in the lower extremities. Regarding the central nervous system, clinical presentation includes ischemic strokes due to endothelial damage, subarachnoid haemorrhage, vision loss caused by ischemic damage of the optic nerve, and paralysis of any of the cranial nerves.^15^

The treatment of this condition is based on the comorbidities of the patients, because if the diagnosis is made when the patient is over 65 years, or either has heart or renal failure (this understood as a creatinine >1.7 mg/dL), nervous or gastrointestinal system compromise, it is considered a patient with poor prognosis with a high risk of mortality within the first year.^17,18^ According to the American College of Rheumatology/Vasculitis Foundation Guideline for the Management of Antineutrophil Cytoplasmic Antibody–Associated,^20^ management of the asthma symptoms should be address based on the *Global Initiative for Asthma (GINA).*^19^ Regarding the disease, in patients with an active and severe condition, rituximab should be considered the first line treatment, over other drugs like cyclophosphamide. Glucocorticoids (GC) can be used in high dose either intravenous (IV) or oral as part of the initial therapy, but the evidence showed if a biological therapy is included, reduced dose of GC can be administered with a great clinical outcome.^19,20^ If the patient is still in remission after the management, therapies can include rituximab, methotrexate, azathioprine, mycophenolate mofetil or leflunomide. If the condition is not severe, low-dose GC are the pillar of the treatment, either alone or with combination with any of the previously mentioned drugs.^20^ Mepolizumab, previously used in flares or refractory disease,^8^ is only now used in patients with active non-severe eGPA alone or could be added to other immunosuppressor agents in case of relapsing symptoms^10^. In patients with alveolar haemorrhage, the implementation of plasma exchange was dismissed in the last European Alliance of Associations for Rheumatology (EULAR) meeting.

Finally, the approach for the management of this condition must be done by a multidisciplinary team because the impact of this condition in the quality life affects the patients’ physical and mental health which can be modified with close follow-ups, physical and pulmonary therapy, as well as psychological accompaniment, leading to an improvement in the quality life of these patients as well as the therapeutic adherence.^21^

## CONCLUSIONS

eGPA is a low-incidence condition with different phenotypes; therefore, the clinical features can be either usual or with an insidious clinical display, leading to a challenging diagnosis. In patients out of the common settings of the diagnosis for this condition, if there is any chance the patient could have the disease, it should be considered, because the main factor to improve the clinical outcome of this condition is the early start of the treatment, moreover, impacting in the quality life of the patients.
